# *Escherichia coli* has an undiscovered ability to inhibit the growth of both Gram-negative and Gram-positive bacteria

**DOI:** 10.1038/s41598-024-57996-x

**Published:** 2024-03-28

**Authors:** Ertan Kastrat, Hai-Ping Cheng

**Affiliations:** 1 Department of Biological Sciences, Lehman College, City University of New York, Bronx, NY 10468 USA; 2 The Graduate Center, City University of New York, New York, NY 10016 USA

**Keywords:** Drug discovery, Microbiology

## Abstract

The ability for bacteria to form boundaries between neighboring colonies as the result of intra-species inhibition has been described for a limited number of species. Here, we report that intra-species inhibition is more common than previously recognized. We demonstrated that swimming colonies of four *Escherichia coli* strains and six other bacteria form inhibitory zones between colonies, which is not caused by nutrient depletion. This phenomenon was similarly observed with non-flagellated bacteria. We developed a square-streaking pattern assay which revealed that *Escherichia coli* BW25113 inhibits the growth of other *E. coli,* and surprisingly, other Gram-positive and negative bacteria, including multi-drug resistant clinical isolates. Altogether, our findings demonstrate intra-species inhibition is common and might be used by *E. coli* to inhibit other bacteria. Our findings raise the possibility for a common mechanism shared across bacteria for intra-species inhibition. This can be further explored for a potential new class of antibiotics.

## Introduction

Bacteria are known to produce compounds to inhibit the growth of microbial competitors in their environment^1^. This inhibition is typically mediated through the production of broad-spectrum secondary metabolites and narrow-spectrum peptides, such as bacteriocins and microcins, aiding bacteria in their competition against other species and strains^2–4^. Border formation between colonies is a peculiar type of competition, where bacteria within a colony collectively inhibit the growth and expansion of an encroaching neighboring colony, resulting in the formation of a cell-free or low cell density border between the colonies. Inter-strain inhibition between swarming bacterial colonies has been well described, using terms such as border formation, kin discrimination/recognition, mutual inhibition, and demarcation line formation, to describe the formation of an inhibitory boundary observed between encroaching colonies of *Bacillus subtilis, E. coli, Pseudomonas aeruginosa, Proteus mirabilis,* and *Myxococcus xanthus*, mediated by cell-contact dependent killing and cell surface receptors involved in distinguishing strain identities^5–14^.

Competition is not limited to genetically distinct bacteria, as intra-strain killing has been described in the literature. In some reports, subpopulations of bacteria within a strain experience differential gene expression mediated by quorum signaling, causing a subset of the population not involved in the quorum response to undergo programmed cell death, resulting in cell lysis^15–18^. This behavior is believed to result in an overall gain of fitness for the population through the release of virulence factors from lysed cells. Intra-strain border formation between colonies has been poorly described in the literature and has not been assessed for non-flagellated bacteria. Intra-strain border formation between swarming colonies of *Paenibacillus dendritiformis* is the best studied example in the literature. Border formation between streaks of *P. dendritiformis* occurs when the neighboring inoculant is of the same morphotype and is exacerbated through the addition of antibiotics and by lower concentrations of peptone present in the growth medium^19^. This inhibition was found to be associated with a 12 kDa narrow-spectrum protein secreted into the environment, termed sibling lethal factor (Slf), resulting in the lysis of bacteria on the colony front challenged with a neighboring inoculant^20,21^. The inhibition phenotype is only observable when challenged with a neighboring colony present at a short distance, as this allows for the above-threshold accumulation between the growths of a secreted protease and the Slf precursor protein. The protease, subtilisin Carlsberg, initially serves as a growth promoting factor, but at higher concentrations, as is found between colony fronts, it is associated with growth inhibition. Inhibition occurs through the proteolytic cleavage of the Slf precursor protein by subtilisin, resulting in the Slf-mediated lysis of *P. dendritiformis*^20^*. Paenibacillus dendritiformis* cells residing farther from the colony front, but still adjacent to the neighboring colony, are exposed to sub-lethal concentrations of Slf. These cells undergo a phenotypic change where rod-shaped cells become cocci, which are resistant to the lytic protein. Intra-strain border formation has also been observed for swarming colonies of *B. subtilis, E. coli, Salmonella typhimurium,* and *Pseudoalteromonas haloplanktis*, which interestingly contradicts other reports concluding border formation only occurs across strains^5,13,22^. Border formation between genetically identical colonies spreading on the surface was postulated to form due to biophysical factors such as the diffusion rate of nutrients, propagation speed of colonies, and agar concentration, conclusions supported by experimental and mathematical modeling. The authors believe there may be a common genetic mechanism in bacteria to regulate flagellar motility in response to these biophysical factors, causing a slowing down of bacterial motility until propagation of the colony front ceases, creating the visible borders between colonies. An alternative hypothesis based on experimental and mathematical modeling of intra-strain border formation in *B. subtilis* suggests biochemical factors play a role, though the exact cause of this repulsion, whether it is due to a secreted inhibitor, remains unknown^23^. The authors found agar concentration and initial inoculation distance of neighboring colonies determines whether an inhibitory border forms or if the colonies fuse. A mathematical model taking bacterial death rate into account was used to suggest border formation is due to the presence of an inhibitory compound. Seemingly contradictory data are presented in the literature regarding the presence or absence, and cause, of intra-strain border formation.

Here, we describe the phenomenon of intra-strain inhibition between swimming clonal bacterial colonies. The phenotype is represented by the formation of a low cell density border between encroaching clonal colony fronts. We show that this appears to be a widespread phenomenon found in genetically diverse bacterial species, including Gram+ , Gram-, and for the first time, in non-flagellated bacteria. Using *Escherichia coli* BW25113 as our model, our results suggest this inhibition is mediated by a diffusible compound, and surprisingly, observed *E. coli* inhibiting other Gram+ and Gram-bacteria, including multi-drug resistant clinical isolates. *Escherichia coli* mediated broad-spectrum inhibition of other species has not been described in the literature. Our results suggest intra-strain competition may be mediated by a mechanism common to all bacteria, possibly functioning in bacterial population control. Additionally, the inhibitory compound leads to bacterial membrane damage and cell death, suggesting it is bactericidal in nature. Identification of the active compound could lead to the development of new antibiotics.

## Results

### Formation of an inhibitory border between swimming clonal inoculants

Our initial serendipitous observation of an inhibitory border forming between swimming bacterial colony fronts expanding through 0.3% motility agar was first seen with *Sinorhizobium meliloti* Rm1021. We were screening for motility in *S. meliloti* mutants and had left Petri dishes on the bench at room temperature for some weeks. Upon inspection, we noticed the swimming colonies had formed a visible boundary with their neighboring colonies. The expected radial expansion of the colonies did not occur, as we observed colonies forming a square shape. The inhibitory border was not cell-free, but rather harbored a lower cell density compared to regions where opposing colony fronts did not meet.

**Figure 1:**
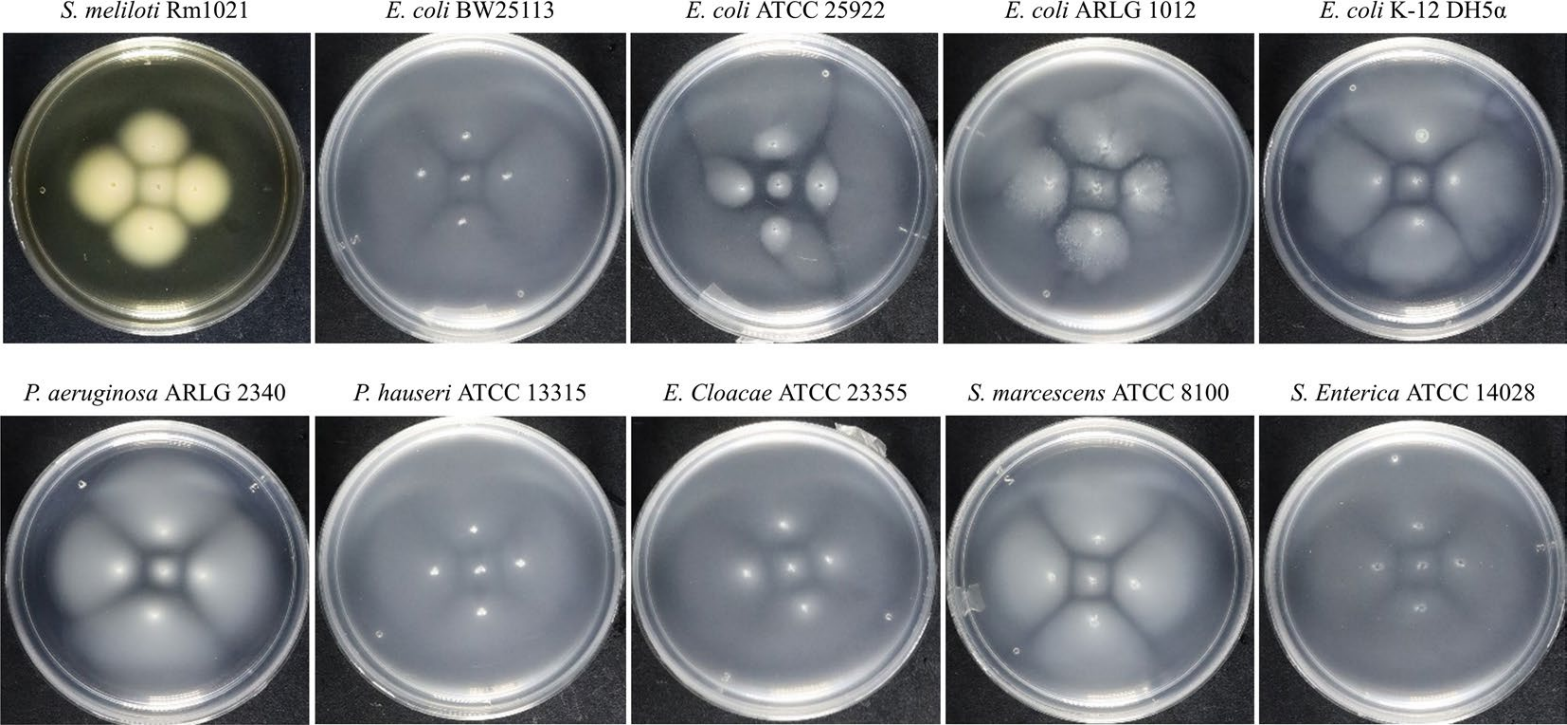
Formation of a low cell density border between clonal colony fronts during swimming motility. Bacteria from the same colony were inoculated at 5 points in media containing 0.3% agar and incubated for 24 h at 37 °C, except for *S. meliloti*, which was incubated for 48 h at 30°C. All strains were inoculated in low nutrient media consisting of 10% LB (0.1% tryptone and 0.05% yeast extract), except for *S. meliloti*, which was inoculated in the nutrient rich medium, LBMC. An inhibitory border between colony fronts is observed, with the strongest effect seen with the center inoculant. Cells were inoculated in a quincunx pattern with each point being 1 cm from the center inoculant.

We then decided to screen for the prevalence and ubiquity of intra-strain inhibition between swimming colonies of flagellated bacteria (Table 1) using *S. meliloti,* four strains of *E. coli, Pseudomonas aeruginosa*, *Proteus hauseri*, *Enterobacter cloacae*, *Serratia marcescens*, and *Salmonella enterica*, to reproduce our initial observations, with some modification. We inoculated the flagellated bacteria in 0.3% motility agar in a quincunx pattern to visually exacerbate inhibitory border formation (Fig. 1). We chose this pattern as it exacerbates the inhibitory effect on the center inoculant, leading to the formation of a deformed square-shaped colony with smaller size than the surrounding colonies. All five inoculation points used bacteria from the same colony to account for genetic variation between inoculants. All bacteria we screened produced an inhibitory boundary when challenged with a neighboring colony. The degree of inhibition appears to vary, as some bacteria display inter-colony borders composed of lower cell density, while in other cases, the borders appear less pronounced due to an increased concentration of cells in the region. The most prominent inhibitory effect is seen with the center inoculant, as its colony is inhibited on four fronts, resulting in a swimming colony with a square shape. Inoculating bacteria in low nutrient media enhanced the appearance of the inhibitory border compared to rich media (data not shown), though this was not required for border formation. Our data suggest there is a common mechanism of intra-strain inhibition in bacteria. This inhibition may be due to an extracellular compound or may be cell-contact dependent.

**Table 1:** Strains used.

Bacteria	Key characteristics	Source
*Acinetobacter baumannii* ARLG 1783	MDR clinical isolate	ARLG*
*Enterobacter cloacae* ATCC 23355	Flagellated. Cephalosporinase +	ATCC**
*Escherichia coli* ARLG 1012	Flagellated. MDR clinical isolate	ARLG
*Escherichia coli* BW25113	Flagellated. Parent strain for Keio knockout collection	CGSC***
*Escherichia coli JW5437-1 ∆rpoS*	rpoS746(del)::kan. Keio knockout collection. Flagellated	CGSC
*Escherichia coli JW2662-1 ∆luxS*	luxS768(del)::kan. Keio knockout collection. Flagellated	CGSC
*Escherichia coli* DH5α	Derived from K-12 strain. Flagellated	Ref^62^
*Escherichia coli* ATCC 25922	Uropathogenic clinical isolate. Flagellated	ATCC
*Klebsiella pneumoniae* ARLG 1002	MDR clinical isolate	ARLG
*Pseudomonas aeruginosa* ARLG 2340	Flagellated. MDR clinical isolate	ARLG
*Pseudomonas aeruginosa* ATCC 27853	Flagellated. Strain: Boston 41501	ATCC
*Proteus hauseri* ATCC 13315	Originally deposited as *P. vulgaris.* Flagellated	ATCC
*Salmonella enterica* ATCC 14028	Subspecies *enterica* serovar Typhimurium. Flagellated	ATCC
*Serratia marcescens* ATCC 8100	Non-pigmented. Flagellated	ATCC
*Sinorhizobium meliloti* Rm1021	Wild type strain. Flagellated	Ref^63^
*Staphylococcus aureus* ARLG 1574	MDR clinical isolate	ARLG
*Staphylococcus aureus* ATCC 25923	Strain: Seattle 1945	ATCC

*ARLG (Antibiotic Resistance Leadership Group).

**ATCC (American Type Culture Collection).

***CGSC (Coli Genetic Stock Center, Yale University).

### Inhibition is not cell-contact dependent and is enhanced by the age of bacterial colonies

To assess if the inhibition we observed was the result of a compound released by bacteria as opposed to being a form of cell-contact dependent inhibition, we modified the swimming inoculation pattern by spotting and square streaking bacteria onto LB containing 0.8% agar to determine if intra-strain inhibition can occur across a distance. We chose to inoculate bacteria in a square streak pattern to represent the 4 surrounding inoculants from our swimming assays. We also chose a spotting pattern to represent the center inoculant from our previous assay, allowing us to inoculate in the center of and outside the square streak to compare the degree of inhibition at different positions. Bacteria spotted in the center would theoretically be exposed to the highest concentration of compounds secreted by surrounding bacteria due to multiple merging diffusion fronts of the surrounding square streak. This concept is similar to placing multiple antibiotic infused paper disks onto the agar in a similar pattern and observing stronger inhibition in regions where diffusion fronts of the drugs combine and intersect. Five spotted inoculants were added at the same time (+ 0 h) as the square streak was produced, or added after 24, 48, or 72 h following initial incubation of the square streak, to assess the effect of colony age on the degree of inhibition. We postulated that if inhibition is mediated by a diffusible compound, incubation of the square streak prior to spotting the bacteria should result in a stronger inhibitory effect as the inhibitor would accumulate in the growth medium over time. Four spot inoculants were added near each outer edge of the square streak (left, top, right, bottom; 0.5 cm, 1.0 cm, 1.5 cm, 2.0 cm from the square streak’s outer edge, respectively), with the fifth spotted in the center of the square streak, 2.0 cm from the square streak’s inner edge. Any variation in degree of inhibition between the center and surrounding inoculants could then be attributed to the merging of diffusion fronts, which may carry an inhibitory compound, resulting in a stronger inhibitory effect.

**Figure 2:**
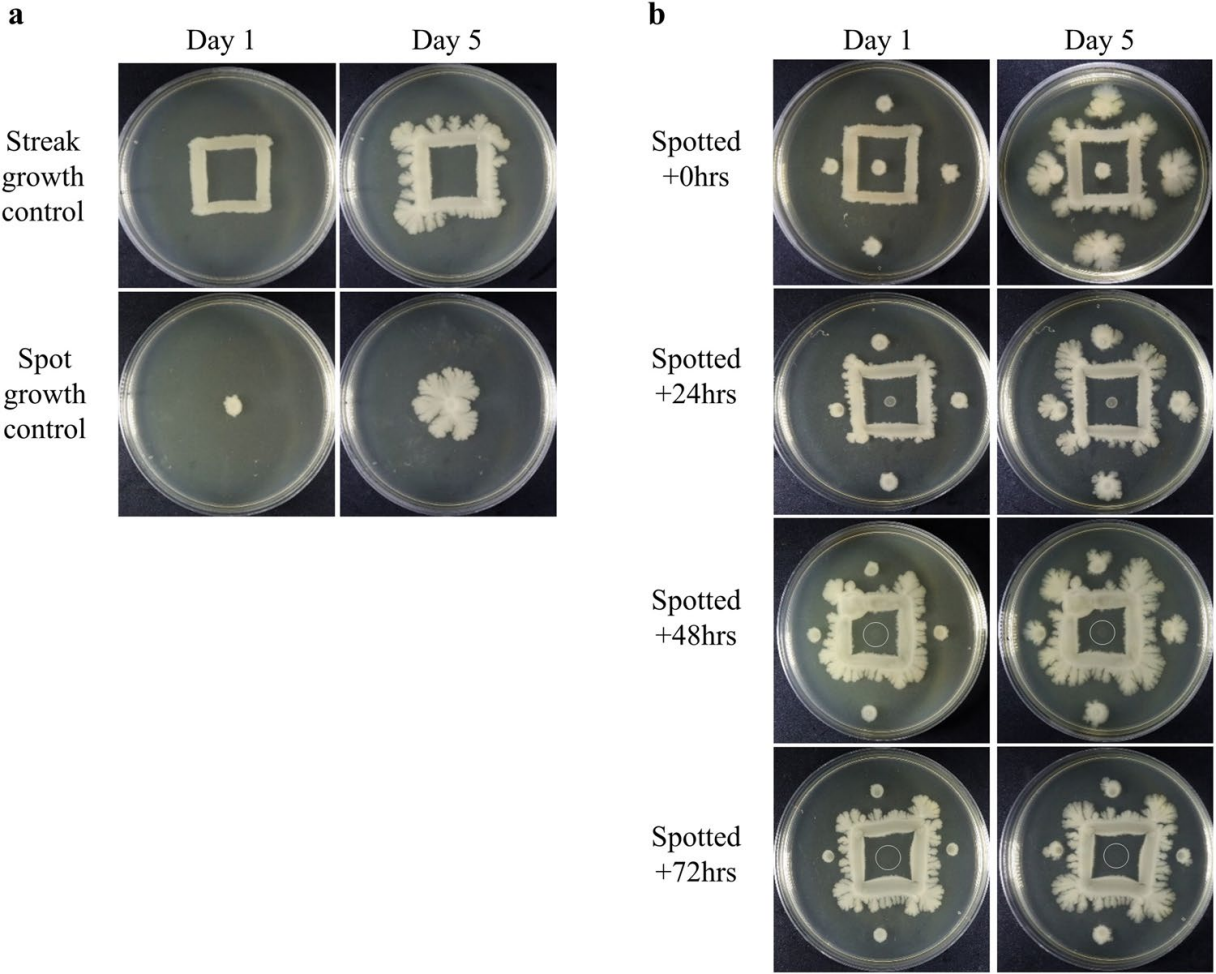
Inhibitory effect observed is independent of cell contact. *Escherichia coli* BW25113 was streaked in a square pattern and spotted on LB containing 0.8% agar. Cells streaked in a square pattern exhibit outward growth but no inward expansion (**a**, top row). The spot inoculant grows outward, uninhibited (**a**, bottom row). Images were taken at Day 1 and Day 5 of incubation. The two inoculation methods were combined by incubating the square streak for 0, 24, 48, or 72 h, followed by spotting an inoculant on 5 points at a 0.5 cm increment, beginning from the left of the square streak, going clockwise distance (0.5 cm on the left to 2.0 cm below the square streak) (**b**). Images were taken at Day 1 and Day 5 of incubation after spotting. Longer preincubation of the square streak results in stronger inhibition of the spotted inoculants, with the stronger effect seen with the center inoculant. Central inoculant spotting location highlighted for clarity (**b**, circles drawn on 48- and 72-h images).

As controls, we produced a square streak or placed a single spot inoculant of *E. coli* BW25113 onto LB 0.8% agar. When streaked in a square pattern, we observed continued outgrowths of the square streak, but no inward growth into the untapped nutrient rich region, over a 5-day period (Fig. 2a, top row). This suggested the inner region of the square streak may possess a comparatively higher concentration of an inhibitor, suppressing the growth of bacteria into this nutrient-rich region. Without contestation of neighboring bacteria, a single spotted inoculant grew radially, as expected, producing a large colony (Fig. 2a, bottom row).

We then spotted *E. coli* at the same time as the square streak was produced (Fig. 2b, Top row). All 5 spotted colonies initially appeared to grow to a similar density after 1 day of incubation. However, the surrounding inoculants continued to grow over a 5-day period while the center inoculant’s growth was inhibited after the first day of incubation. Additionally, bacteria spotted outside the square streak preferentially grew away from the neighboring square streak; this effect was slightly reduced as distance between the two inoculants increased. Preincubation of the square streak for 24, 48, or 72 h prior to spotting resulted in surrounding spotted colonies that were similar in size compared to those inoculated simultaneously with the square streak for the first day of incubation. However, deviation in size of surrounding spotted inoculants at the end of their 5-day incubation period was observed, where colonies spotted at earlier timepoints were larger than those spotted after preincubation of the square streak (Fig. 2b, descending rows, Day 5 column). In comparison to the surrounding inoculants, the center inoculant’s growth was inhibited to a greater degree as prior incubation time of the square streak increased (Fig. 2b, descending rows). No growth was visually observable with the center inoculant when the square streak was preincubated for 72 h (Fig. 2b, bottom row). In all cases, the center inoculant does not appear to grow past the first day of incubation, while the surrounding spotted inoculants continue to grow. This suggests inhibition is not merely due to nutrient depletion, as bacteria can continue to grow, and it is likely an inhibitory compound is accumulating in the growth medium over time, leading to regulation of total biomass on the growth medium.

Together, our data suggest inhibition is mediated by a diffusible compound and that concentration of the inhibitor increases over time. The data also suggest nutrient limitation is unlikely the sole cause of growth inhibition, as even with 8 days of total incubation time (72 h preincubation of the square streak followed by 5 additional days of growth, Fig. 2b, bottom row), surrounding colonies continue to grow while no growth is observed with the center inoculant following 1 day of incubation.

### Non-flagellated bacteria also exhibit intra-strain inhibition

**Figure 3:**
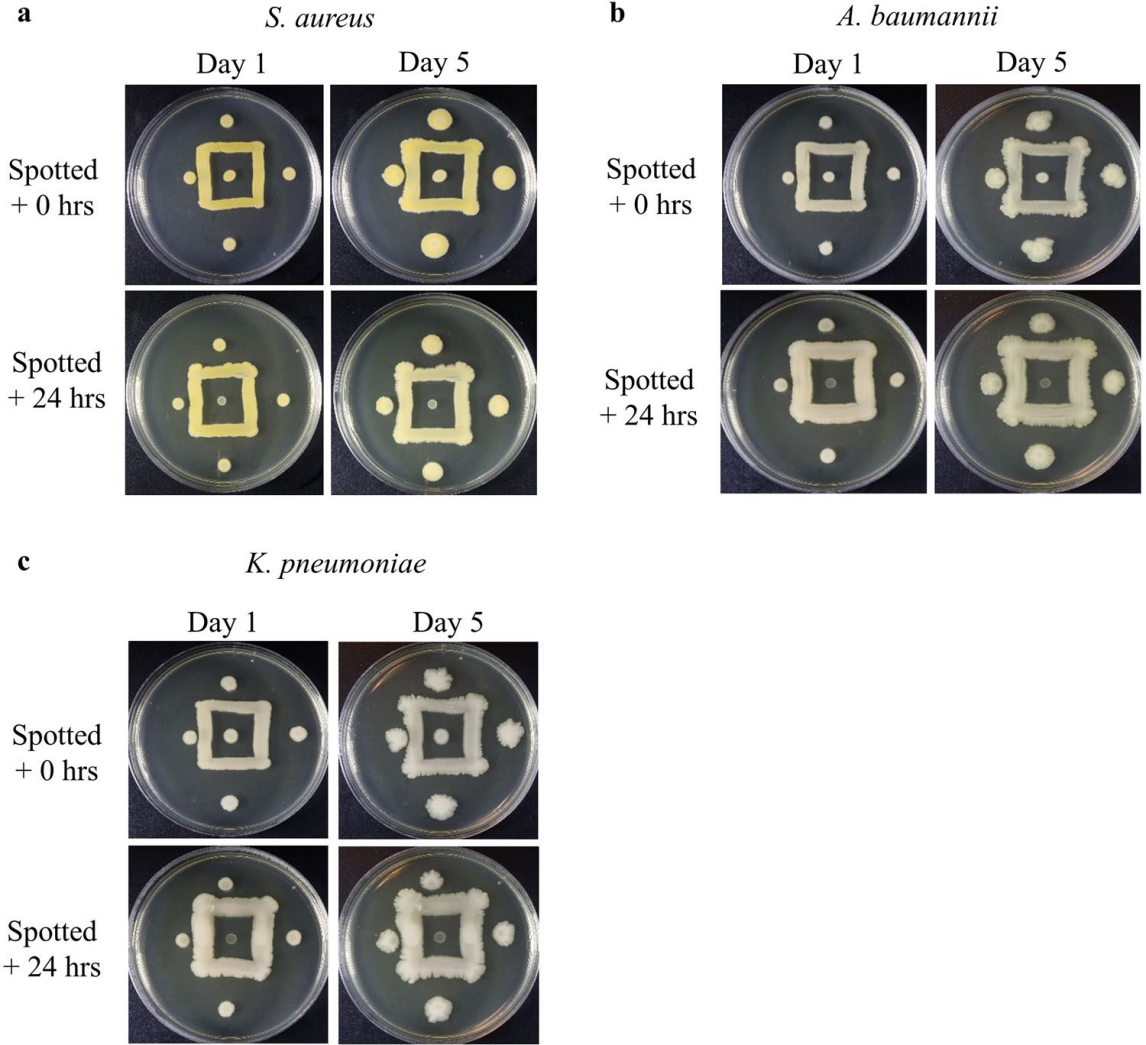
Inhibition screening of non-flagellated bacteria. Non-flagellated, multi-drug resistant clinical isolates, *S. aureus* ARLG 1574 (**a**), *A. baumannii* ARLG 1783 (**b**), and *K. pneumoniae* ARLG 1002 (**c**) exhibited the same inhibition phenotype as *E. coli* BW25113. Inhibition of the center inoculant is strongest and displays no visible change in growth over a period of 5 days. Deformation of colony fronts between the square streak and spotted inoculants are visible and most obvious when observing the closest spotted inoculant (left of streak) at Day 5.

Intra-strain inhibition between colonies of non-flagellated bacteria has not been previously described in the literature. To address this and to further expand our assessment on the ubiquity of this phenomenon, we repeated our square streak and spotting inoculation pattern using non-flagellated bacteria. We used multi-drug resistant clinical isolates of *Staphylococcus aureus, Acinetobacter baumannii,* and *Klebsiella pneumoniae* (Table 1). We observed the same inhibition phenotype previously seen with flagellated bacteria with the non-flagellated bacteria we used (Fig. 3). Deformation of expanding colony fronts can be seen, most notably between the left spotted inoculant and the neighboring square streak, and consistent with previous results, the strongest inhibition was observed with the center-spotted inoculant (Fig. 3a–c, Day 5). Together, our results suggest intra-strain inhibition may be ubiquitous, as it was seen in flagellated, non-flagellated, Gram-positive, and Gram-negative bacteria. Additionally, the data suggest inhibition of growth and border formation is not dependent on the presence of flagella, as the same inhibition pattern appears when non-flagellated cells are used. We cannot rule out that deformation of colony expansion is not a chemotactic response, as flagellar-independent motility, such as twitching, gliding, spreading, or darting, may at least be partially responsible for deviation from radial colony expansion^24–26^. However, our strongest evidence of chemically mediated growth-inhibition is displayed by the growth inhibition of inoculants spotted in the center of a square streak. This suggests it is unlikely biophysical factors are the cause of our observed phenotype and lends credibility towards the hypothesis that border formation is mediated by a diffusible inhibitor.

### Inhibition phenotype is independent of quorum signaling and the stress response

**Figure 4:**
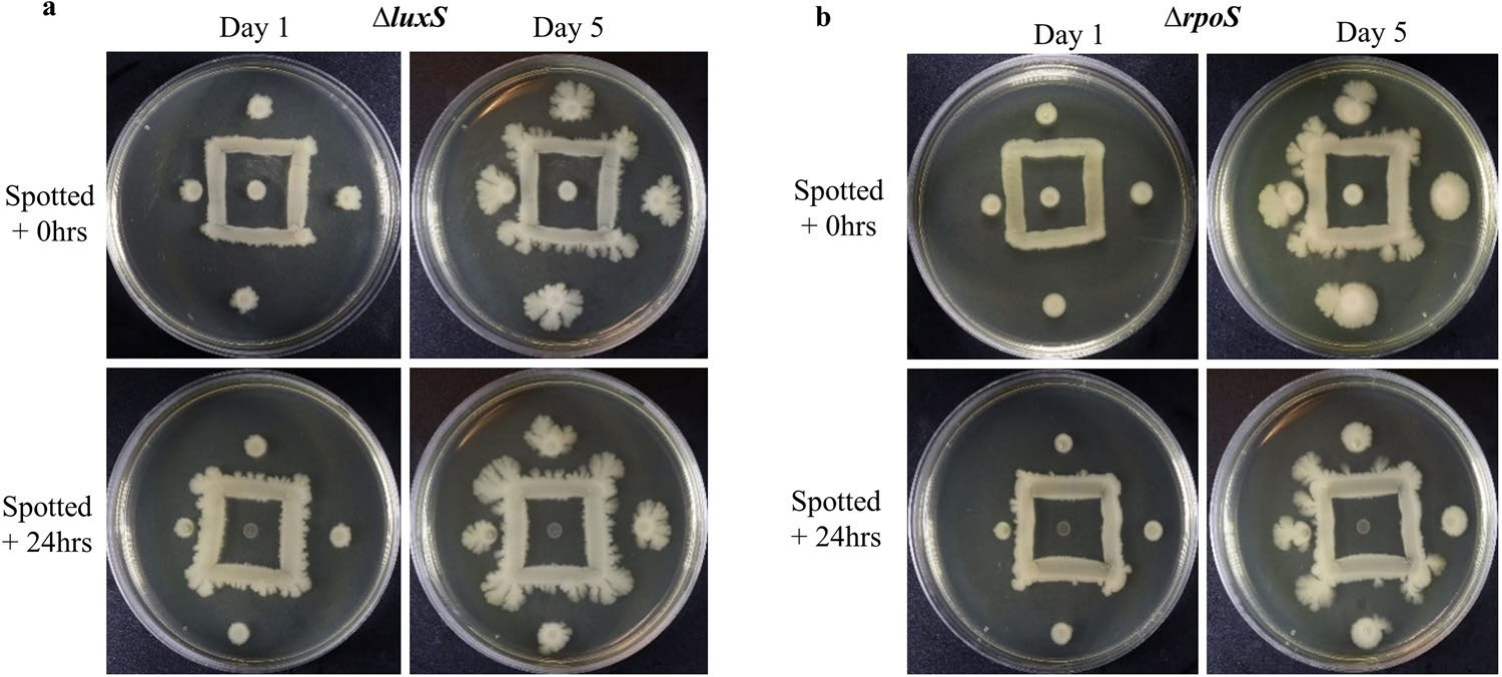
Inhibition is not regulated by quorum sensing or the stress response. *Escherichia coli* Keio collection knockouts in quorum sensing (*ΔluxS*) and the stress response (*ΔrpoS)* exhibit an identical inhibition phenotype as the parent strain BW25113, seen in Fig. 2.

Based on our previous observations showing a positive correlation between colony age and the strength of inhibition, we assumed transcriptional changes associated with quorum signaling or the stress response may play a role in the inhibition phenotype we observed. Therefore, we decided to assess if LuxS-mediated quorum signaling, or the RpoS-mediated stress response, were involved in the production of the inhibitory compound. ∆*luxS* and ∆*rpoS* Keio collection mutants from *E. coli* BW25113 parent strain did not produce an altered phenotype when compared with our previous data (Fig. 4). Our results show the inhibition phenotype is independent of quorum signaling and the RpoS-mediated stress response. This suggests inhibitor production may occur at a constant basal rate or may require specific conditions to elicit upregulation and production of the active compound.

### Growth inhibition is not due to nutrient depletion

**Figure 5:**
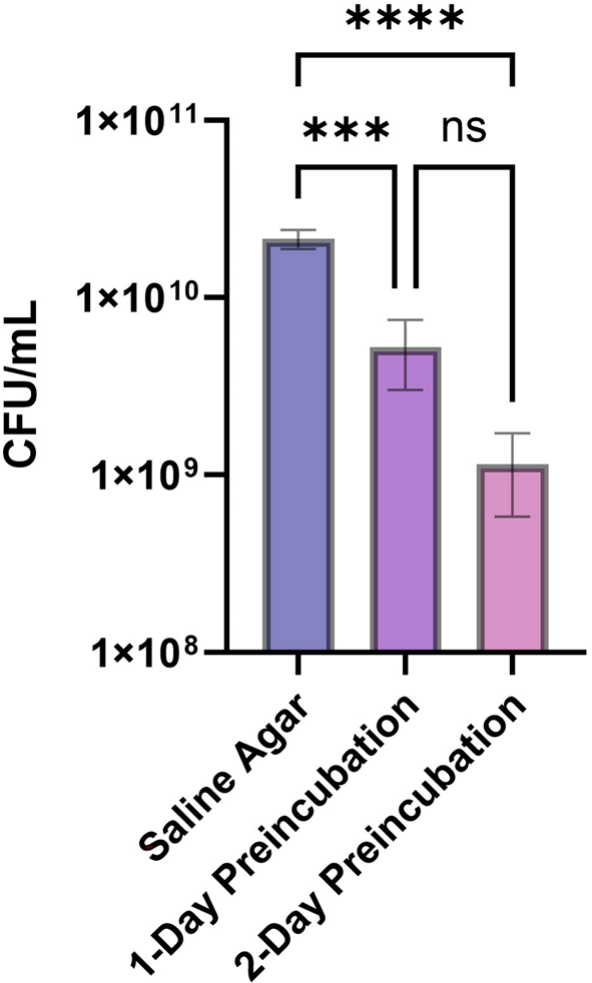
Inhibition of growth for spotted center inoculant is not due to a starvation effect. *Escherichia coli* BW25113 was spotted on a saline agar plate, or on LB in the center of a square streak of *E. coli* BW25113 previously incubated for 1 or 2 days. Spotted inoculants were incubated for 24 h. Significant reduction in CFU is observed when spotted inoculants are added to plates containing the square bacterial streak (as seen in Fig. 2) previously incubated for 1 (****P = 0.0002) and 2 days (***P < 0.0001). ns = not significant values are means from three replicates.

To rule out the possibility of the observed growth inhibition resulting from nutrient depletion or starvation, we spotted *E. coli* BW25113 onto nutrient-free saline agar and compared CFU to bacteria spotted on nutrient rich media in the center of a 24-h and 48-h preincubated square streak. We determined CFU 24 h after incubation of the spotted inoculants and observed a reduction in CFU for inoculants added to the center of a streak preincubated for 1 day (*P* = 0.0002) and 2 days (*P* < 0.0001) (Fig. 5) compared to cells spotted onto nutrient-free saline. Our data suggest that the inhibitory effect we observed cannot be explained by a lack of nutrients and that the active compound may function through a bactericidal mechanism of action due to the reduction in viable bacterial cells.

**Figure 6:**
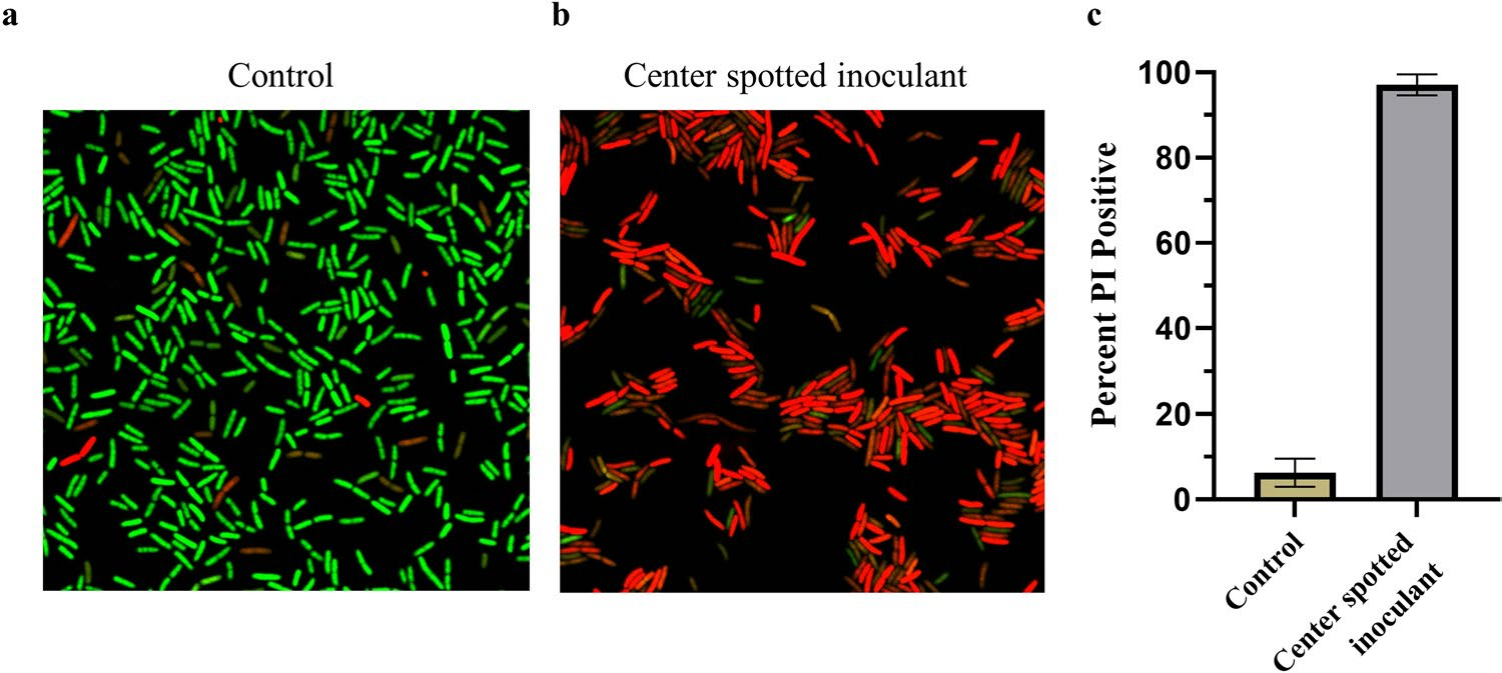
Diffusible inhibitory compound causes damage to the bacterial cell membrane. *Escherichia coli* BW25113 was spotted onto an LB 0.8% agar (**a**, control) or in the center of a 1-day preincubated square streak of BW25113 (**b**). Plates were then incubated for 90 min and stained with cell permeable SYTO9 (green, live cells) and cell impermeable propidium iodide (PI, red cells, damaged membrane). Percentage of PI positive cells was determined from three independent fields of view from each condition (**c**).

To gain preliminary insight into the mechanism of action for the inhibitory compound, we stained *E. coli* BW25113 with nucleic acid stains using cell permeable SYTO9 and cell impermeable Propidium Iodide (PI) to determine if membrane damage was occurring. We spotted bacteria onto sterile LB agar as our control to compare with cells spotted in the center of a 24-h preincubated square streak, followed by 90 min of incubation prior to staining. We observed that most of the cells spotted in the center of the square streak exhibited red fluorescence, indicating damage to the bacterial membrane (Fig. 6). Taken together, our data suggest a diffusible compound is released by *E. coli* and causes cell death in bacteria of the same strain. Based on our previous observations, this mechanism may be ubiquitous amongst bacteria.

### Escherichia coli inhibits the growth of other bacteria

**Figure 7:**
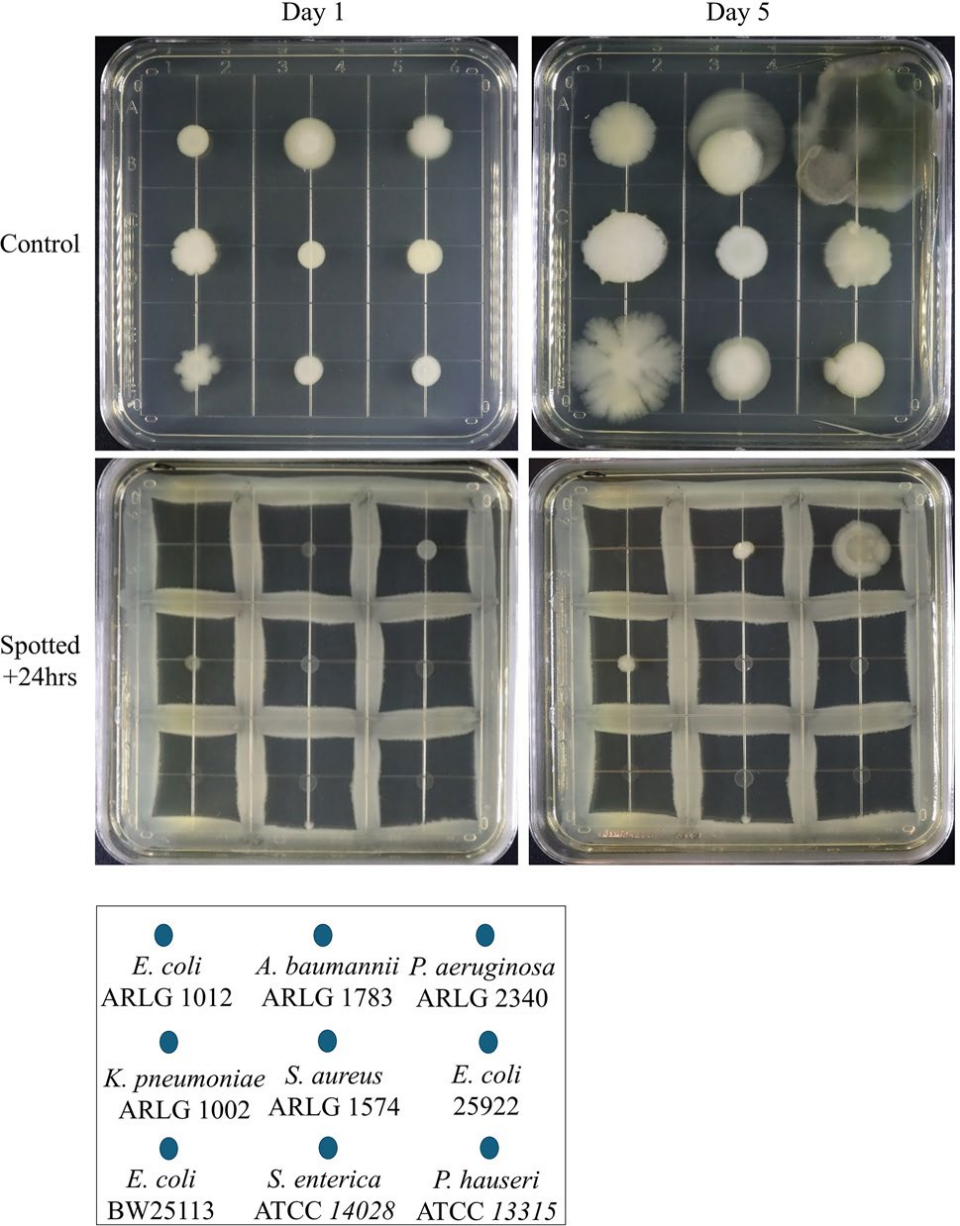
Inhibitory activity observed across bacterial species. *Escherichia coli* BW25113 was streaked in a grid pattern and incubated for 24 h. Spotted inoculants, including multi-drug resistant clinical isolates, were added in-between *E. coli* streaks, incubated, and imaged for an additional 5 days with the streaked grid, or onto a plate with no grid streaked, as a control. Growth of all bacteria spotted onto the preincubated *E. coli* grid plate was inhibited to varying degrees.

Having demonstrated that intra-strain inhibition can be observed in diverse bacterial species, we were curious to see if this inhibitory effect was limited to within a strain and if it was a possibility that there could be a conserved mechanism in bacteria regulating this process. If this is a conserved mechanism common to all bacteria, cross-species inhibition may be observed. We used *E. coli* BW25113 as the producer strain for the proposed inhibitory compound. We streaked *E. coli* in a 3 × 3 grid pattern and incubated the plate for 24 h. Following incubation, we spotted different strains of *E. coli* and other bacteria, some of which included multi-drug resistant clinical isolates (Table 1), in the center region of each grid (Fig. 7). As controls, we spotted these same bacteria onto a fresh LB agar to serve as a comparison for growth inhibition. Compared to controls, we observed varying degrees of growth inhibition against all spotted inoculants. Our data suggest a diffusible inhibitory compound from *E. coli* has a broad spectrum of activity, affecting self and non-self. These data show *E.* coli can surprisingly inhibit the growth, to varying degrees, of other bacteria, suggesting a novel antibiotic compound could be derived from *E. coli*. The data suggest our simple and reproducible methods of screening could be used to discover novel antibiotic agents from other bacteria. We also reveal that one of the best-studied microorganisms, *E. coli*, can surprisingly exhibit broad-spectrum inhibitory activity against other bacteria.

## Discussion

One of the reasons proposed to explain the lack of progress towards novel antibiotic discovery over the past few decades is the idea that we have overmined antibiotics from culturable microorganisms^27^. Though drug discovery using traditional screening methods has slowed, it remains as a viable option allowing us to screen for bacterial competitive interactions that may lead to novel drug discoveries^28,29^. The difficulty in finding new antibiotic compounds has sparked the development of novel and complex techniques, such as culturing classically unculturable bacteria, high-throughput screening of drug libraries, activation of silent biosynthetic gene clusters, and artificial intelligence guided drug design, to screen for new drug candidates^30–37^. Inspired by our initial observation of inhibition between clonal bacterial swimming colonies, we developed a simple method to screen bacteria for the production of inhibitory compounds which may not be detectable using typical screening methods. Our observations suggest some of the most well-studied organisms in bacteriology are producing inhibitory compounds not previously described. This may be a fundamental and conserved mechanism involved in bacterial population control which can limit the growth of self and non-self.

Boundary formation in swimming *E. coli* colonies was previously described and found to be attributed to genetic variation within a population leading to inter-strain but not intra-strain boundary formation^13^. Our work contrasts that finding, as we observed intra-strain boundary formation, even when using inoculants from a single colony (Fig. 1). Border formation due to inhibition between different swarming strains has been described for *P. mirabilis*, *M. xanthus*, *P. aeruginosa*, and *B. subtilis*, and is found to be cell-contact dependent^8,10–12,14^. In our work, we found border formation can result from intra-strain inhibition independent of cell contact (Figs. 2 and 3). Reports in the literature describe border formation between swarming *P. dendritiformis* colonies that was found to be mediated by a narrow-spectrum secreted protein, mediating intra-strain inhibition^19,20,38^. We find our work to be different from what has been reported in the literature, as we observed cross-species and cross-strain inhibition (Fig. 7) mediated by *E. coli*, the phenotype of which, to the best of our knowledge, has not been described in the literature. We also note that the self-inhibition phenotype occurs over larger distance, contrasting with what has described for *P. dendritiformis*^20^. Together, this suggests our observed phenotype is mediated by an undiscovered and undescribed compound.

Another report of intra-species boundary formation suggests the phenotype is due to a conserved genetic mechanism involved in controlling the flagellar response, contrasting with our observations where we show inhibition occurring over a distance, even in non-flagellated bacteria (Figs. 2 and 3)^22^. One study suggests border formation may be due to an inhibitory compound produced by bacteria, and found that agar concentration and initial inoculation distance affect whether or not intra-strain boundary formation occurs, where agar concentrations < 0.5% resulted in merging^23^. This contrasts with our finding of swimming inhibition in 0.3% agar but supports our conclusion that inhibition is mediated by a secreted compound (Fig. 1).

We postulate the variation of experimental conclusions amongst different groups could be due to an inhibitor being produced at low concentrations, resulting in a phenotype that is difficult to observe. We presume the inhibitory compound is produced at basal levels because inhibition is stronger with longer preincubation times of an initial streak (Fig. 2). Basal-level transcription could explain the weak inhibitory border formation amongst swimming inoculants which traverse distances at a faster pace compared to distances traversed by surface inoculants. The slower spread of bacteria on the surface allows for accumulation of the inhibitory compound which can then exert its effects at a greater distance from the bacterial source. The inoculation patterns we chose exacerbate the inhibition phenotype, possibly resulting in the merging of multiple inhibitor diffusion fronts, or, from overcrowding or exposure to the inhibitory compound leading to autoinduction of inhibitor production^39^. Additionally, inhibitory activity is not dependent on quorum sensing nor the RpoS-mediated stress response (Fig. 4), which are pathways known to induce antibiotic production^40–42^. This suggests the active compound may be produced by a silent biosynthetic gene cluster which could be upregulated by finding the appropriate eliciting conditions^43^. It is likely that a compound with no obvious resistance in producer strains would only be secreted under specific conditions and at very low concentrations, otherwise, it would not allow for bacterial growth to initially occur. The question remains as to why would a bacteria inhibit itself and why would this mechanism be common. We postulate this may occur to ensure bacterial biodiversity in the environment, as it will not allow a particular species to gain an advantage to become the predominant microbe in an environment inhabited by other microorganisms, many of which are dependent on each other for their growth.

We were able to rule out that inhibition of growth was solely due to nutrient starvation by comparing CFU of bacteria spotted on nutrient-free agar against bacteria spotted in the center of a sqaure streak (Fig. 5). Additionally, we observed significant membrane damage with SYTO9/PI staining of centrally spotted inoculants (Fig. 6). A robust PI signal response required 90 min of exposure in the center streak. In the process of spotting cells, it is quite likely that the inhibitory metabolite on the agar surface is diluted when adding a liquid drop of bacteria. Then, it would be expected for there to be a delay in a positive signal for membrane damage, as it would take time for the compound to diffuse from the surrounding media into the diluted region. Membrane permeability to PI has also been found to occur following treatment with non-membrane targeting bactericidal antibiotics in similar timeframes, leaving this as an alternative hypothesis for our data interpretation^44^.

It is possible the inhibition we observed within and across bacterial strains and species is the result of multiple compounds, or it may be due to a single compound produced by many bacteria utilizing a common biosynthetic pathway. Judging by the consistent pattern of inhibition, the latter scenario seems more likely to us, where a single or family of similar compounds with slight modifications are produced by bacteria to regulate growth in crowded conditions. The phenotype of self-inhibition raises a concern about the clinical significance of such a finding if inhibition is non-specific in nature. Conversely, this may be a positive as there is no obvious genetic predisposition to resistance in cases where the producer strain is killed by the active compound it produces. Not harboring resistance does not necessarily indicate the inhibitor is non-specific in action, as such drugs have recently been discovered^31,37,45–47^.

One method we are currently exploring to aid in our discovery of an active compound involves excision of the inter-colony inhibitory region from the agar, followed by assessing conditions for chemical extraction and concentration of bioactive compounds contained within. We hope this will result in the recovery of a supernatant with self and broad-spectrum antibiotic activity. The difficulty here is that obtaining an adequate volume of extract using this method may be impractical for further studies. Our data suggest the inhibition-mediating compound may be produced at a low concentration, as the effect is only observable when using certain growth patterns. Our future studies are focused on finding optimal conditions to elicit the production of the compound mediating the observed growth inhibition; this will allow us to perform further characterization and eventual identification with scaled-up production. Adjusting the nutrient profiles of growth media, investigating the effect bacterial interactions have on enhancement of our observed phenotype, and using known inducers of bacterial secondary metabolite production, such as ultra-low concentrations of antibiotics, are methods we are currently exploring to aid in our discovery^28,43,48,49^. While we cannot completely rule out that the phenotype we observed is solely due to metabolic waste product accumulation, we hope that future studies aimed at identifying the active compound will provide further insight. Based on the literature, it seems unlikely our observation is simply due to the accumulation of waste or extremes of pH, as the literature shows such factors play larger roles in the dense center of a bacterial colony, with these effects greatly minimized near the colony fronts or at a distance^50,51^. It has been reported that *E. coli* colonies can continue to expand up to 130 h after inoculation, whereupon metabolic waste products likely limit further growth^52^. This time point is far later than the time it takes to observe the phenotype we observed, suggesting this inhibition is mediated by an unreported mechanism. This same work has also briefly reported on the ability of a mature *E. coli* colony to inhibit itself and *Proteus*, postulated to occur through a secreted inhibitor, though further work has not been published^52^. Therefore, we propose that the inhibitory effect we observe between bacterial colonies of the same species and that which is observed with *E. coli* against other bacteria is mediated by an undiscovered compound. We assume that investigation into this phenomenon was overlooked due to the specific method required for observing the phenotype and that the active compound is likely produced at low concentrations, especially since it inhibits the growth of the producer, resulting in the compound being masked by more abundant metabolites during metabolome mining.

Our findings are surprising, as we observed inhibition within strains, across strains, and across species, and against multi-drug resistant clinical isolates, using *E. coli* BW25113 as our producer strain (Fig. 7). *Escherichia coli,* one of the most well-studied organisms, is not known to produce broad-spectrum antibiotic compounds*. Escherichia coli* does produce bacteriocins and microcins, which have a narrow spectrum of activity and are transcribed in conjunction with antitoxins, conferring resistance to producer strain^53–56^. Our observations suggest bacteria secrete a diffusible inhibitory compound into their surroundings which can limit the growth of clonal cells and other bacteria. This novel finding is in contrast to the protective effects *E. coli* can exhibit when cocultured with other bacterial species, as was shown with multi-species biofilms, where a protective effect is exerted between bacterial species against environmental insults^57–59^. We postulate the difference in the killing effect we observe versus the protective effect reported is due to the timing of coculturing, as our data suggests the time allotted for growth prior to introducing a new inoculant significanly alters the inhibition phenotype (Fig. 2b). Thus, cocultures do not allow for the accumulation of an inhibitory compound to biologically revelant levels. What we observed is that a higher density inoculum is required to significantly inhibit a fresh inoculum of far lower density. This idea holds true in the observation of inhibition at the lower cell density colony fronts seen in our swimming assays (Fig. 1) and with the spotted inoculants surrounding the square streak, where inhibiton occurs at the intercolony fronts (Figs. 2, 3 and 4). We believe this is due the inhibitor being expressed at a basal level, which can only be evidenced when a large number of bacteria, as seen in a colony, inhibits the growth of the low desnity colony front, or of a freshly spotted inoculant. This reasoning justifies as to why *E. coli* does not immediately self-inhibit when spotted onto a plate. Similarly, the density of bacteria per volume in culture compared to colonies on agar plates is orders of magnitude lower. The idea of an inhibitor being produced at basal levels is supported by the literature, as bacterial antibiotic production is typically controlled by biosynthetic gene clusters (BGCs), many of which are silently expressed^60,61^.

Our screening for inhibitory activity amongst genetically diverse bacteria suggests self-inhibition is a widespread mechanism (Figs. 1, 2 and 3). The data also suggest intra-strain competition may be an evolutionary conserved or convergent mechanism, as varying degrees of inhibition can be observed across species (Fig. 7), suggesting crosstalk between the pathways involved in mediating intra-strain competition. We found the inhibition phenotype to be associated with membrane damage and that it is independent of nutrient availability, quorum sensing, and the RpoS-mediated stress response (Figs. 4, 5 and 6). If the genes involved in inhibitor production are found and can be mutagenized or if the inhibitor can be neutralized, it could serve as a method to increase biomass and production for industrial fermentation. From a clinical perspective, identification of the active compound and mechanism of action may lead to the development of new drugs or identification of novel targets for antibiotics.

## Methods

### Culture conditions and media

LB (Luria–Bertani) medium was used for culturing all bacteria, except *S. meliloti* and *S. aureus*. LB supplemented with 2.5 mM MgSO4 and 2.5 mM CaCl_2_ (LBMC) was used for culturing *S. meliloti*. Trypticase Soy media was used for culturing *S. aureus*. Agar concentrations were 1.5% for solid medium, 0.3% for swimming assay, and 0.8% for surface inhibition assays. All strains from Table 1 were grown aerobically at 37 °C, except for *S. meliloti*, which was similarly grown, at 30 °C. Colonies for inoculation into broth were selected from freshly streaked plates and mid-logarithmic phase bacteria were subcultured into fresh medium at a concentration of 10^7^ CFU/mL. Liquid cultures were agitated at 200 rpm.

### Inhibition of clonal swimming colonies

Bacterial cells were transferred to swimming plates in a pattern corresponding to the 4 vertices of a square 1 cm apart, with a 5th inoculant added to the center using a sterile toothpick in a 35 mm × 10 mm Petri dish. *Sinorhizobium meliloti* was inoculated in LBMC with 0.3% agar. All other strains were inoculated in LB 0.3% agar with reduced concentrations of tryptone (0.1%) and yeast extract (0.05%). *Sinorhizobium meliloti* swimming colonies were imaged 48 h after incubation at 30 °C. All other bacteria were imaged 24 h after incubation at 37 °C.

### Surface growth inhibition

For intra-strain surface inhibition, exponential phase bacterial cultures were streaked in a square (2 cm × 2 cm) onto a 100 mm round Petri dish using sterile cotton swabs. Plates were then spotted with 2 μL of 5*10^7^ CFU/mL of exponential phase culture arranged 0.5, 1.0, 1.5, and 2.0 cm (Left, Top, Right, Bottom, respectively) from the edge of the square streak, with a 5th inoculant added to the center region of the square streak. Bacterial cultures were spotted at the same time point (+ 0 h) or at 24, 48, or 72 h after incubation of the square streak. All plates were incubated at 37 °C in a sealed container and imaged daily for 5 days. Similarly, *E. coli* BW25113 streaked as the producer strain in a 3 × 3 grid pattern on a 100mm square Petri dish for broad spectrum inhibition assay.

### CFU comparison of inoculants spotted in the square streak

Similar to the method described above, *E. coli* BW25113 was streaked onto LB in a square pattern and then was spotted in the center 24- or 48-h after incubation of the streak. An inoculant (2 μL of 5*10^7^ CFU/mL ) was also spotted on cell-free agar containing only 0.85% NaCl as a control. Spotted plates were incubated at 37 °C for 24 h. CFU was determined by cutting out a thin layer where the inoculant was spotted, placing it in 100 μL of LB broth, and vortexing it rigorously for 10 s. 50 μL of the suspension was removed, serially diluted, and plated onto LB 1.5% agar and incubated at 37 °C for 24 h. Mean CFU for each condition was calculated from 3 replicates. Statistical analysis was done by using 1-way ANOVA followed by Tukey’s test using GraphPad Prism.

### Live/dead staining and confocal imaging

Integrity of *E. coli* BW25113 cells inhibited by other *E. coli* BW25113 inside a square steak was determined using Live/Dead BacLight Bacterial Viability Kit (L13152, Sigma) with some modification. After a 24-h incubation, *E. coli* BW25113 cells (2 μL of 5*10^7^ CFU/mL) were spotted in the center of the square streak and incubated at 37 °C for 90 min or onto fresh LB 0.8% agar as a control. The spotted *E. coli* BW25113 cells were stained by adding 10 μL of 2 × concentration of SYTO9 (12 μM) and Propidium Iodide (60 μM) on the spotted bacterial cells and incubated at 21 °C in the dark for 15 min. This process was repeated one more time to ensure sufficient staining. The agar with stained inoculant was cut from the plate and placed inverted in a glass bottom petri dish (P50G-1.5–30-F, Matek) so that spotted bacterial cells are facing the glass bottom. Bacterial cells were imaged on a Leica SP5 laser confocal microscope using a 63 × water immersion objective (HCX PLO APO CS 1.20 NA), 3 × digital zoom, and sequential scanning at 488 nm/500–530 nm (excitation/emission) for SYTO9 and 543 nm/604–700 nm Propidium Iodide.

### Image analysis

Confocal images of live/dead staining were analyzed using Cell Profiler version 4.2.5 (cellprofiler.org) to determine the percentage of PI positive cells. Images were converted to grayscale using the “ColorToGray” module and cells were counted using the “IdentifyPrimaryObjects” module. Object identification parameters used advanced settings with the lower threshold of object diameter set to the measured pixel width of the smallest bacterial cell. Cells outside of this range and near the border of the image were discarded. A global threshold strategy utilizing the Otsu method with 3 classes was employed. This process was repeated with the “ColorToGray” module employed to split the RGB image into independent channels and only converting objects with a red signal into grayscale, followed by cell counting with the parameters set above. This ensures any cell with red signal (PI stain) will be counted, as cells can contain a mixture of both dyes to indicate cells with varying degrees of membrane damage. Cell counts from 3 fields of view for each condition were obtained, and percent PI positive cells was calculated.

## Data Availability

All data generated or analysed during this study are included in this published article.
